# Determinants of Production Efficiency of Maize-Dominated Farmers in Western Parts of Ethiopia in Gudeya Bila District: Evidence under Shifting Cultivation Area

**DOI:** 10.1155/2022/3355224

**Published:** 2022-04-26

**Authors:** Tolesa Tesema

**Affiliations:** Department of Agricultural Economics, Wollega University, Nekemte, Ethiopia

## Abstract

**Background:**

In Ethiopia, maize is produced as major food crop that is based on traditional methods of production, and there exists inefficiency in the use of available scarce resources. Thus, poor people are failing to achieve rapid economic growth, development, and food security still today in the country. Hence, the best possible means of achieving development is through increasing the production efficiency of farmers. Thus, to estimate the levels of production efficiency, this study specifically used only data of farmers who are producing without ploughing by oxen and without using fertilizers in the study area under shifting cultivation.

**Method:**

Stochastic frontier production is used to estimate the technical efficiency score, and the cost frontier model is used to estimate production efficiency. To determine the determinants of production efficiency, the Tobit model was used in this study.

**Result:**

The Tobit model results show that loss due to wild animals, experience of household, and off-farm income had a negative impact on production efficiency of farmers. Regarding the positive determinants of production efficiency, land conservation practice and mobile use have a positive influence. *Conclusion and recommendation.* The farmers in the study area are inefficient in the production of maize. Since the loss of maize products is high due to wild animals such as pigs, apes, and monkeys that results in production inefficiency, the agricultural policies and strategies of Ethiopian governments should be directed toward providing tourism to protect those wild animals. Additionally, to increase the production efficiency, construction of terraces and soil bunds to conserve land and supporting the farmers by providing network facilities for mobile usage that boost maize production efficiency of farmers is essential for policymakers.

## 1. Introduction

Ethiopia's economy is heavily reliant on agriculture, which accounts for 40% of GDP, 80% of exports, and over 75% of the country's employment [1]. It is Ethiopia's primary means of reducing poverty, and it provides a solution for long-term development by enhancing the standard of living for disadvantaged rural areas and boosting farmers' incomes [2]. However, farming techniques have been changed little over the centuries yielding low outputs and making farmers vulnerable to the effects of unpredictable weather patterns [3]. In cognizant of these, Ethiopia's government has established a new 10-year development plan that will run from 2020/21 to 2029/30, with the goal of maintaining the country's outstanding growth attained under the growth and transformation plans. However, the risk of falling back into poverty remains considerable, especially for rural households reliant on rainy agriculture [4]. Furthermore, Ethiopia is one of the most inefficient agricultural producers in the world, owing to an ever-increasing population growth rate that is unable to meet the needs of the local community [5]. The major food crops produced in almost all regions of the country have variation in the volume of production across the regions attributed to the extent of area devoted to each crop type, weather change, and a shift in preference for the crops grown [6]. In Ethiopia, over 90% of farmers are smallholders cultivating one hectare or less of land and farming techniques have changed little over the centuries, yielding low outputs and making farmers vulnerable to the effects of unpredictable weather patterns [7]. Even if maize is an important food crop in the low land of Gudeya Bila districts, disseminating improved technologies is difficult in the area. Thus, its productivity remains below its potential; hence, the community may not educate their children and there may be no health improvements and no life sustenance and an increase in the price of the commodity that results in severe underdevelopment and food insecurity. Maize yield gaps were also linked to efficiency, resource, and technological yield gaps, with the components of the yield gap varying by maize variety, soil type, year, and farming system [8]. Because of the scarcity of land and low quality of organic fertilizer, improving the efficiency of farmers increases production, ensures food security, and protects the environment [9]. Consequently, if farmers are producing to supply the surplus to the market after feeding themselves with reducing land per capita due to population growth, they need to adopt new farming practices and hence increase their efficiency, and food aid from developed countries can be decreased [10–13]. However, the efficiency study in one farming system does not represent the farming system in another place, because social growth is dynamic. The key gap in this study is that the farming system in the study area is under shifting cultivation, with an emphasis on farmers who produce maize without using oxen and simply plant maize seed on the soil. The farmers studied in this research work are not using fertilizers and improved seeds. This shows that the inputs that farmers used in the study area are different from other areas. Moreover, during the night, farmers spend their effort on the protection of crops from wild animals such as pigs in addition to daytime duties such as clearing land for maize production and threshing. As a result, this farming method differs from the methods studied by other scholars. Given agricultural production stagnation, the measurement of efficiency differences is attributable to a variety of conditions, and the diversity of surroundings in which farmers operate does not have to be unique to sub-Saharan Africa. Thus, efficiency analysis in such a farming system shows that the efficiency of smallholder farmers requires greater government commitment because the dissemination of technology is difficult in such a farming system because the area is not suitable for using technology. Thus, this study analyzed the levels of production efficiency and identified the determinants of production efficiency by adding new variables such as loss due to wild animals such as pigs, apes, and monkeys and use of mobile for information transmission between the farmers and government agency as additional determinant variables of production efficiency over past scholars.

## 2. Methods

### 2.1. Description of the Study Area

This study was carried out in western parts of Ethiopian low lands of Gudeya Bila districts under shifting cultivation only. The farming system in the district is mainly mixed crop-livestock production in which livestock provides a source of manure for crops and the residual from crop output was used as a source of feeding for livestock. Agriculture is mainly characterized by a rain-fed production system which is used for livelihood sustenance. Maize is one of the major cereals grown in the study area where farming is mostly with limited oxen and no mechanized farming system is available. The samples that were included in this study were only the farmers who are producing under shifting cultivation and who are not using the oxen to know their production efficiency. Those farmers who produce with oxen are not included in this sample.

### 2.2. Design of Sampling Techniques and Questionaries

Multistage sampling techniques were used in this study. In the first stage, Gudeya Bila was selected from western parts of Ethiopia since maize was the dominant crop in the district and the researcher is convenient to the study area. In the second stage, three kebeles namely Gubin, Sawa, and Maja were purposively selected from the district since they are low land of the district and the farming system in those kebeles are based on shifting cultivation which was quite different from other. What makes this research different from others is it covers only the household that produces without ploughing oxen and also not applying the fertilizer on their farmland. Finally, 154 households that produce maize were selected by probability proportional to the sample size in the lowlands of districts based on the below formula of Yamane.

The sample size was determined based on the following formula given by Yamane [14]:

(1) $$\begin{array}{c} n=\frac{N}{1+\left(e^{2}\right)N\;},\; \end{array}$$

where *n* is the sample size, *N* is the number of maize-producing households which was 8765, and e is the desired level of precision which was taken to be 8%.

This research employed a cross-sectional survey using a structured questionnaire which was distributed to the sample household to collect amounts of output produced and amounts of inputs used such as land, labor (during day and night time), and seeds; the socioeconomic, demographic, institutional, and farm-specific characteristics, and cost of input incurred for maize production such as cost of labor used, rental cost of land, and cost seed.

### 2.3. Model Specification for Efficiency and Determinants of Efficiency

The stochastic frontier model was employed to estimate the parameters of production function and the level of efficiency. This is because of the fact that this technique accounts for measuring inefficiency factors and technical errors occurring during measurement and observation [15]. To take into account the effects of these errors, the stochastic frontier model was used in this study. Following Aigner et al. [16] and Meeusen and van Den Broeck [17], the stochastic frontier model defined below was adopted for this study:

(2) $$\begin{array}{c} Y_{i}=f\left(X_{i};\beta \right)+v_{i}-u_{i}, \end{array}$$

where *Y*_i_ measures the quantity of maize output of the *i*^th^ farm in the Gudeya Bila district, *X*_i_ is the vector of input variables used by the *i*^th^ farmer in the lowland area such as land, labor, and seed used by the sample household.

*β* is the vector of unknown parameters. The functional specification *f*(*X*_*i*_; *β*) is a proper production function (Cobb–Douglas). The disturbance term *v*_*i*_is intended to capture the effects of the stochastic noise and it is assumed to be *v*_*i*_∼N (0,*δ*^2^). The disturbance, *u*_*i*_, captures the technical inefficiencies.

Production efficiency from the stochastic production frontier was regressed using a censored Tobit model on farm-specific independent variables that shows disparities in efficiency across farms. Tobit regression [18] is specified as:

(3) $$\begin{array}{c} E*=\delta o+\delta_{m}Z_{im}+\mu , \end{array}$$

(4) $$\begin{array}{c} \frac{v}{z}\approx \;normal\left(0,\delta^{2}\right), \end{array}$$

where *E∗*_is a_ latent variable representing the efficiency scores of maize producer farmers. *δ*_is_ a vector of unknown parameter to be estimated. *Z*_*im*__is_ a vector of explanatory variables *m* (*m* = 1,2,…,n) for farm household such as X1 = amount off loss due to wild animals such as apes, pigs, and monkeys during preharvest of maize. X2 = education levels in year of schooling. X3 = family size in number. X4 = sex of household in dummy (1 if male, 0 if female heeded household). X5 = farm size in hectare. X6 = livestock holding in tropical livestock unit. X7 = slope of land measured in dummy (1 if flat and zero if steep). X8 = distance to plot in minute. X9 = land conservation, (one for plot which is conserved, zero otherwise). X10 = distance to the markets market in minute. X11 = extension contacts in number of contacts during production season. X12 = use of radio measured dummy (one if farmers use radio, zero if not). X13 = use of mobile measured as dummy (1 if farmers used mobile, 0 if not). X14 = credit use measured in dummy (one if farmers received credit, zero if not). X15 = off-farm income dummy (one if farmers engaged in off-farm activities, zero if not). *μ* is the error term that is independently and normally distributed with zero mean and variance *δ*^2^.

Denoting *E*_*i*_ as observed variables,

(5) $$\begin{array}{c} E_{i}=\left\{\begin{array}{cc} 1, & \text{if }E_{i}{}^{*}>=1,\\ 0, & \text{if }E_{i}{}^{*}<=0, \end{array}\right\}. \end{array}$$

Following McDonald and Moffitt [19] from the likelihood function decomposition of marginal effects, the two-limit Tobit model is as follows:

The unconditional expected value of the dependent variable is given by

(6) $$\begin{array}{c} \frac{\partial E\left(y\right)}{\partial X_{j}}=\left[\varphi \left(Z_{u}\right)-\varphi \left(Z_{L}\right)\right].\frac{\partial E\left(y^{*}\right)}{\partial X_{j}}+\frac{\partial \left[\varphi \left(Z_{u}\right)-\varphi \left(Z_{L}\right)\right]}{\partial X_{j}}+\frac{\partial \left[1-\varphi \left(Z_{u}\right)\right]}{\partial X_{j}}. \end{array}$$

The expected value of the dependent variable conditional upon being between the limits is given by

(7) $$\begin{array}{c} \frac{\partial E\left(Y^{*}\right)}{\partial X_{j}}=\beta m.\left[1+\frac{\left\{Z_{L}\phi \left(Z_{L}\right)-Z_{u}\phi \left(Z_{u}\right)\right\}}{\left\{\varphi \left(Z_{u}\right)-\varphi \left(Z_{L}\right)\right\}}\right]-\left[\frac{{\left\{\phi \left(Z_{L}\right)-\phi \left(Z_{u}\right)\right\}}^{2}}{{\left\{\varphi \left(Z_{u}\right)-\varphi \left(Z_{L}\right)\right\}}^{2}}\right]. \end{array}$$

The probability of being between the limits is given by

(8) $$\begin{array}{c} \frac{\partial \left[\varphi \left(Z_{u}\right)-\varphi \left(Z_{L}\right)\right]}{\partial X_{j}}=\frac{\beta_{m}}{\sigma }\left[\phi \left(Z_{L}\right)-\phi \left(Z_{u}\right)\right], \end{array}$$

where *φ*(.) is the cumulative normal distribution, ∅(.) is the normal density function, *Z*_*L*_ = −(*X*_*i*_′*β*/*σ*)and *Z*_*u*_ = (1 − *X*_*i*_′*β*/1) are standardized variables that came from the likelihood function given the limits of y^*∗*^, and *σ* is the standard deviation of the model.

## 3. Results

### 3.1. Estimation of Production Functions

Of the total variables considered in the production function, three of them (seed, land, and labor) had a significant effect that makes a difference in the productivity of maize. The coefficients of the production function are interpreted as elasticity. Hence, the elasticities of output to seed, land, and labor are 0.38, 0.18, and 0.16, respectively, suggesting that maize production was more sensitive to seed. As a result, a 1% increase in the amount of seed will result in a 0.38% increase in maize production, keeping other factors constant. The scale coefficient was calculated to be 0.72 indicating decreasing returns to scale (Table 1). This implies that there is potential for maize producers to continue to swell their production because they are in stage II of the production surface, where resource use and production are believed to be normally utilized.

### 3.2. Estimation of Stochastic Cost Function

The dual cost function derived analytically from the stochastic production function is given as follows:

(9) $$\begin{array}{c} \mathrm{lCmi}=8\text{.}37+\text{0.}32\text{cost output}+\text{0.}34\text{cos seed}+\text{0.}34\text{cost land}+\text{0.}022\text{cost labor,} \end{array}$$

where *lCmi* is the cost of producing maize and *i* refers to the *i*^th^ sample household.  Table 2 shows the estimation of cost functions.

This result shows that if the cost of inputs such as seeds and land increases, the cost of outputs also increases at five percent levels of significance. The coefficient shows that as the cost of seed increases by one birr, the cost of outputs increases by 0.32 percent and as the cost of labor increases by one birr, the ease of the cost of outputs is 0.34 percent. Farmers estimate the amounts for cost of output depending on the cost of inputs they apply.

### 3.3. Production Efficiency Score

The result of the frontier model revealed that farmers in the study area were relatively low in production efficiency. As illustrated in the above Table 3, the production efficiency level of sample households was 36% with minimum and maximum efficiency scores of 1.78% and 79.7%, respectively. This is due to the reason that those farmers produce without ploughing by oxen resulting in low levels in terms of techniques of production, hence low production efficiency. The farmers in these research study areas also do not use fertilizers and traditional seeds. Fertilizers and improved seeds are the yield-enhancing technologies but the farmers are yet to apply these technologies because the farming system is not suitable for these technologies that yield low production efficiency. That is, the producer with an average economic efficiency level could reduce the current average cost of production by 64% to achieve the potential minimum cost level without reducing output levels (Table 3).

### 3.4. Determinants of Production Efficiency of Maize-Dominated Farmers and Its Marginal Effects

In the study area, it is common for the loss due to wild animals such as apes, monkeys, and pigs consuming the maize crop in day and night period during the preharvest stage. So, it is difficult for the farmers to control such wild animals even if they devote more time to controlling these wild animals. The coefficient of loss due to wild animals such as pigs, apes, and monkey were statistically significant at 5% and have negative impact on production efficiency of maize under shifting cultivation area. This is due to farmers wasting their time more on protecting wild animals from crops rather than investing their time on weeding the crop that increases its yield. These results of marginal effect show there is a probability of farmers being economically efficient by 0.22%, improvements of mean and economic efficiency by 0.223%, and an overall increase in economic efficiency by 0.108%.  Table 4 shows the determinants of production efficiency and its marginal effects.

The result also indicated that the use of mobile had a positive sign and statistically significant effect on production efficiency at a 10% level of significance. This suggests that on average, households with the use of mobiles exhibit higher levels of production efficiency. This is due to the reason that the use of mobiles allows a household to enhance knowing the production method and information since Ethiopian telecom is disseminating production, price, and rainfall information to the farms through telephone. This result is consistent with that of other studies [20, 21]. The results of the marginal effect indicate that the probability of farmers' allocative efficiency increases by 1.406%, mean allocative efficiency increases by 1.401%, and overall increase in probability and mean efficiency increase by 6.78%.

The sign of land conservation practices such as soil bund, terrace, and tree planting on was a positive effect on production efficiency at a 10% level of significance. These implying farmers who are plotting the maize on conserved land are more efficient than farmers who are plotting on nonconserved land. This could be because the flat conserved land is not susceptible to soil erosion and the fertilizers are not displaced from the sowed place. Few authors [22, 23] found that soil conservation can increase the productivity of farmland in the highland of Ethiopia. Moreover, the results of the marginal effect of the Tobit model shows that the probability of farmers being economically efficient due to conserved land was 0.212% and mean technical and economic efficiency due to conserved land was 0.217% and the overall increase in probability and means efficiency due to conserved land was 0.254%.

In this study, the coefficient of off-farm activity was negatively statistically significant at 5% level of significance effect with respect to economic efficiency. Off/nonfarm activities may affect the efficiency negatively for the reason that the farmers who are engaging in the off-farm activities may shift to the off-farm such as petty trade, handcraft, and carpentry. The result is in line with the findings of a few authors [24, 25]. As computed from the Tobit model, the probability of farmers being economically efficient due to off-farm income earning was 0.03%, the mean economic efficiency increase due to off-farm earning was 0.03%, and the overall increase in probability and mean efficiency was 0.036%.

The experience of the household influenced economic efficiency negatively. This suggested that younger farmers were more efficient than their older counterparts. Older farmers are reluctant to the change of new technology and advancements in techniques of production. These results are in agreement with those of other scholars [26, 27]. The result of the marginal effect after the Tobit model shows the probability of farmers being technically efficient due to an increase in experience of the household was 1.318%, the mean production efficiency increased by 1.72% due to an increase in the experience of household, and the overall increase in probability and mean production efficiency increased by 1.965%.

## 4. Conclusion

The study's principal conclusion is that the efficiency of farmers producing maize without oxen ploughing and under shifting cultivation may be boosted by boosting efficiency and resolving production efficiency problems. Because the farmers are subsistence farmers, using new technologies is costly, and it is difficult to deploy technology in such an agricultural system. Farmers in the study region are inefficient, according to the results of technical allocative and efficiency scores. As a result, agriculture policymakers must work to improve farmer efficiency in the study area. According to the findings of a study on the drivers of production efficiency, the quantity of maize crop loss caused by wild animals such as monkeys, pigs, and apes has a negative association with farmer productivity. Farmers murder wild animals to protect their crops from them, yet by doing so, they are causing the extinction of those species. Thus, providing a controlling mechanism for those animals through tourism is very essential. Secondly, since mobile use has a positive impact on production efficiency, the government of Ethiopia has to provide opportunities for the people who are in the remote area on how they use mobile phones and provide mobile phones at low cost for agricultural information transformation as well as the construct mobile network facilities to the rural communities. Thirdly, since land conservation has positive impacts on production efficiency, improving land productivity status by applying new soil conservation practices on their farm through improved sustainable land management practices especially terrace, planting grass, and soil bund is encouraged especially in the area which is susceptible to soil erosion. Further research has to analyze the comparative production efficiency in the highland, midland, and lowland.

## Figures and Tables

**Table 1:** Estimation of the Cobb–Douglas frontier production function.

Ln output	Coefficient	Standard error	*Z*	*P* value>|z|
Ln of seed	0.38428595^*∗∗∗*^	0.117578	3.27	0.001
Ln of land	0.1887785^*∗∗*^	0.099584	1.9	0.058
Ln of labor	0.16310107^*∗∗∗*^	0.058564	2.78	0.005
Constants	1.5859073^*∗∗∗*^	0.37896	4.18	0
/lnsig2v	−2.34026	0.274023	−8.54	0
/lnsig2u	−1.92611	0.513121	−3.75	0
sigma_v	0.310327	0.042518		
sigma_u	0.381725	0.097935		
sigma2	0.242017	0.056329		
Lambda	1.230072	0.134055		

^*∗*^, ^*∗∗*^, and ^*∗∗∗*^ refers to 10%, 5%, and 1% significance levels, respectively. Source: stochastic frontier model result.

**Table 2:** Estimation of cost functions.

Ln cost of output	Coefficient	Standard error	*Z*	*P* value > *z*
Ln of output	0.47646531^*∗∗∗*^	0.1013794	4.70	0.000
Ln cost of seed	0.32506669^*∗∗*^	0.1603711	2.03	0.043
Ln cost of land	0.34094717^*∗∗*^	0.1341895	−2.54	0.011
Ln cost of labor	0.02229082	0.0804879	−0.28	0.782
Constant	8.3712285^*∗∗∗*^	0.8062753	10.38	0.000
/lnsig2v	−1.413302	0.1241996		
/lnsig2u	−7.619796	67.22597		
sigma_v	0.4932936	0.0306334		
sigma_u	0.0221504	0.7445425		
sigma2	0.2438292	0.0348104		
Lambda	0.0449032	0.757197		

^*∗*^, ^*∗∗*^, and ^*∗∗∗*^ refers to10%, 5%, and 1% significance levels, respectively. Source: stochastic cost frontier model result.

**Table 3:** Technical, allocative, and economic efficiency score.

Variable	Mean	Std. dev.	Min	Max
Production efficiency	0.36	0.14	0.0178	0.797

Source: model result.

**Table 4:** Determinants of production efficiency and its marginal effects.

Determinants of production efficiency	Coefficients	Standard error	Marginal effects		
Education levels	0.00034539	0.0017	0.0007	0.00145	0.00136
Loss due wild animals	−0.00440927^*∗∗*^	0.00261	0.00108	0.00223	0.0022
Family size	0.00295263	0.00286	0.00118	0.00244	0.00233
Sex of household	−0.0003739	0.00162	0.00066	0.00138	0.00129
Farm experience	−0.01753057^*∗*^	0.01965	0.00738	0.0172	0.01318
Family size	0.00816001	0.01214	0.00498	0.01036	0.00978
Land conservation	0.00378686^*∗*^	0.00254	0.00105	0.00217	0.00212
Livestock holding	0.00191826	0.00958	0.00393	0.00817	0.00766
Distance to plot	−0.00018538	0.00076	0.00031	0.00065	0.00061
Slope of land	0.01332231	0.01545	0.00629	0.01322	0.01234
Distance to market	0.00639659	0.01844	0.00774	0.01562	0.01561
Mobile use	0.02443978^*∗*^	0.01645	0.00678	0.01401	0.01406
Radio use	0.0111836	0.01656	0.00685	0.0141	0.01363
Credit use	0.00085454	0.01625	0.00667	0.01387	0.013
Off-farm income	−0.00067308^*∗∗*^	0.00036	0.00015	0.0003	0.0003
Constant	0.74830623^*∗∗∗*^	0.04696			

^*∗*^, ^*∗∗*^, and ^*∗∗∗*^ significant at 10%, 5%, and 1% level significance, respectively. Source: Tobit model results (2021).

## Data Availability

No data were used to support this study.
